# Seroprevalence and durability of antibody responses to AstraZeneca vaccination in Ugandans with prior mild or asymptomatic COVID-19: implications for vaccine policy

**DOI:** 10.3389/fimmu.2023.1183983

**Published:** 2023-05-02

**Authors:** Jennifer Serwanga, Claire Baine, Susan Mugaba, Violet Ankunda, Betty Oliver Auma, Gerald Kevin Oluka, Laban Kato, Isaac Kitabye, Jackson Sembera, Geoffrey Odoch, Peter Ejou, Amina Nalumansi, Ben Gombe, Monica Musenero, Pontiano Kaleebu

**Affiliations:** ^1^ Pathogen Genomics, Phenotype, and Immunity Program, Medical Research Council, Uganda Virus Research Institute and London School of Hygiene and Tropical Medicine, Uganda Research Unit, Entebbe, Uganda; ^2^ Department of Immunology, Uganda Virus Research Institute, Entebbe, Uganda; ^3^ Science, Technology, and Innovation Secretariat, Office of the President, Government of Uganda, Kampala, Uganda

**Keywords:** AstraZeneca vaccine, hybrid immunity, IgG, IgM, and IgA antibody responses, Ugandan population, spike-directed antibodies, nucleoprotein-directed antibodies

## Abstract

**Introduction:**

The duration and timing of immunity conferred by COVID-19 vaccination in sub-Saharan Africa are crucial for guiding pandemic policy interventions, but systematic data for this region is scarce. This study investigated the antibody response after AstraZeneca vaccination in COVID-19 convalescent Ugandans.

**Methods:**

We recruited 86 participants with a previous rt-PCR-confirmed mild or asymptomatic COVID-19 infection and measured the prevalence and levels of spike-directed IgG, IgM, and IgA antibodies at baseline, 14 and 28 days after the first dose (priming), 14 days after the second dose (boosting), and at six- and nine-months post-priming. We also measured the prevalence and levels of nucleoprotein-directed antibodies to assess breakthrough infections.

**Results:**

Within two weeks of priming, vaccination substantially increased the prevalence and concentrations of spike-directed antibodies (p < 0.0001, Wilcoxon signed rank test), with 97.0% and 66% of vaccinated individuals possessing S-IgG and S-IgA antibodies before administering the booster dose. S-IgM prevalence changed marginally after the initial vaccination and barely after the booster, consistent with an already primed immune system. However, we also observed a rise in nucleoprotein seroprevalence, indicative of breakthroughs six months after the initial vaccination.

**Discussion:**

Our results suggest that vaccination of COVID-19 convalescent individuals with the AstraZeneca vaccine induces a robust and differential spike-directed antibody response. The data highlights the value of vaccination as an effective method for inducing immunity in previously infected individuals and the importance of administering two doses to maintain protective immunity. Monitoring anti-spike IgG and IgA when assessing vaccine-induced antibody responses is suggested for this population; assessing S-IgM will underestimate the response. The AstraZeneca vaccine is a valuable tool in the fight against COVID-19. Further research is needed to determine the durability of vaccine-induced immunity and the potential need for booster doses.

## Introduction

It is essential to comprehend the population-specific dynamics of immune responses to SARS-CoV-2 infection and vaccination to assess the vaccine’s effectiveness, the number of booster doses required, and the timing of vaccination post-infection (1). Herd immunity is a viable approach to combating the spread of many infectious diseases, as it may allow for the rapid development of immunity within a population and prevent the vulnerable from encountering the virus (2). As of January 30, 2023, there were 13,168,935,724 vaccine doses administered globally, with 5,493,549,963 people receiving at least one dose and 5,054,793,316 people receiving complete vaccination. According to the most recent data available as of March 25, 2023, 26,406,936 vaccine doses had been administered in Uganda. 19,488,104 individuals received at least one dose of the vaccine, while 13,043,107 individuals, representing 67% of the vaccinated population, were fully vaccinated (https://covid19.who.int/region/afro/country/ug).

Generating effective antiviral antibodies to natural immunity and vaccines requires *de novo* B cell responses to SARS CoV-2. After the initial IgM response, which declines relatively rapidly with viral clearance, class-switched antibodies, predominantly IgG and IgA, are produced (3). These class-switched antibodies are responsible for the protective long-term memory responses against SARS-CoV-2 (4, 5), which enables the body to respond rapidly when re-exposed to an infection. Quantifying the levels of circulating class-switched B cell responses is critical for assessing the state of population-wide immunity and informing strategies for boosting immunity. Prior natural infection studies in this population found that IgG titres persist after acute infections and reflect antibody response magnitude, type, and stability (6). Other studies have also shown that IgG titres remain elevated and relatively stable for months or years following acute infections (7, 8). In both natural infection and vaccinees, rates of antibody decline have been shown to depend on the magnitude of the peak response, the antibody isotypes involved, and the relative contributions of circulating IgG levels (9, 10). When previously infected individuals are immunised, antigen-specific memory B cells are rapidly activated, enhancing their immune efficacy (11). This enables a more rapid and robust response to the antigen than seen in someone who has never encountered it. As new virus variants emerge, protective immunity may get compromised due to critical antigenic mutations in the spike glycoprotein epitopes that may reduce the virus’s recognition by antibodies (12), thereby diminishing the effectiveness of vaccination.

Those previously infected with SARS-CoV-2 have a more rapid and robust antibody response following vaccination than those who have never been infected. Hybrid immunity, elicited by a combination of initial infection and subsequent vaccination, produced antibody levels that were often 1,000-fold higher and with greater neutralisation potency than either natural infection or vaccination alone (13, 14). Due to the increased somatic hypermutation and affinity maturation, mRNA-based vaccines have been shown to elicit antibodies that persist much longer than four months after the booster shot (15). In the absence of prior infection, antibodies induced by the mRNA vaccine provided negligible protection three months after vaccination (16), implying that the vaccine’s elicited immunity was short-lived. In contrast, the durability of hybrid immunity has varied among populations, with some studies reporting the persistence of neutralising antibodies beyond seven months (17). For the viral-vectored AstraZeneca vaccine, hybrid immunity persisted six months after vaccination in some populations (18, 19), and three months in others.

AstraZeneca was the first COVID-19 vaccine to be distributed to most African nations (20). However, the development and evolution of the antibody response elicited by this vaccine in the African setting still need to be clarified. We hypothesised that individuals previously infected with mild and asymptomatic SARS-CoV-2 would produce robust and durable antibody responses in response to vaccination. To test this hypothesis, blood samples were taken at various time points up to one year after vaccination, and ELISA was used to measure the levels of IgG, IgM, and IgA antibodies in the plasma samples.

## Materials and methods

### Study population

This was a prospective study of 86 participants who recovered from mild or asymptomatic primary COVID-19 following rt-PCR-confirmed SARS-CoV-2 infection. All 86 subsequently received both prime and boost doses of the AstraZeneca vaccine and were followed for 12 months after their initial vaccination. Seven individuals who were not observed during the early stages of their infection were omitted from the descriptive analysis. The remaining 79 with available demographic data consisted of 16 females and 63 males with a median age (IQR) of 29 (24–37.5) years. In this study, participants were categorized as asymptomatic or mildly symptomatic based on their hospital records, even though all participants reported experiencing either no or mild symptoms. This classification was used for statistical analysis purposes. Specifically, using clinical records available at the time of infection, 34 individuals were identified as asymptomatic, 13 individuals were found to have mild symptoms, and 32 “mild or asymptomatic” individuals had no symptom records on file. We examined 382 specimens from 86 participants collected between February 24, 2021, and August 3, 2022, to measure the optical densities (ODs) and concentrations of IgG, IgM, and IgA antibodies elicited in response to spike and nucleoprotein antigens over time. The commonly circulating variants at the time of the participants’ infection was the A23.1 variant of interest (21) and the B.1 Lineage (22). All study procedures were approved by the Research and Ethics Committee of the Uganda Virus Research Institute (GC/127/833) and the Uganda National Council for Science and Technology (HS637ES). Participants provided written informed consent to participate in the study.

### Study design

**Figure 1:**
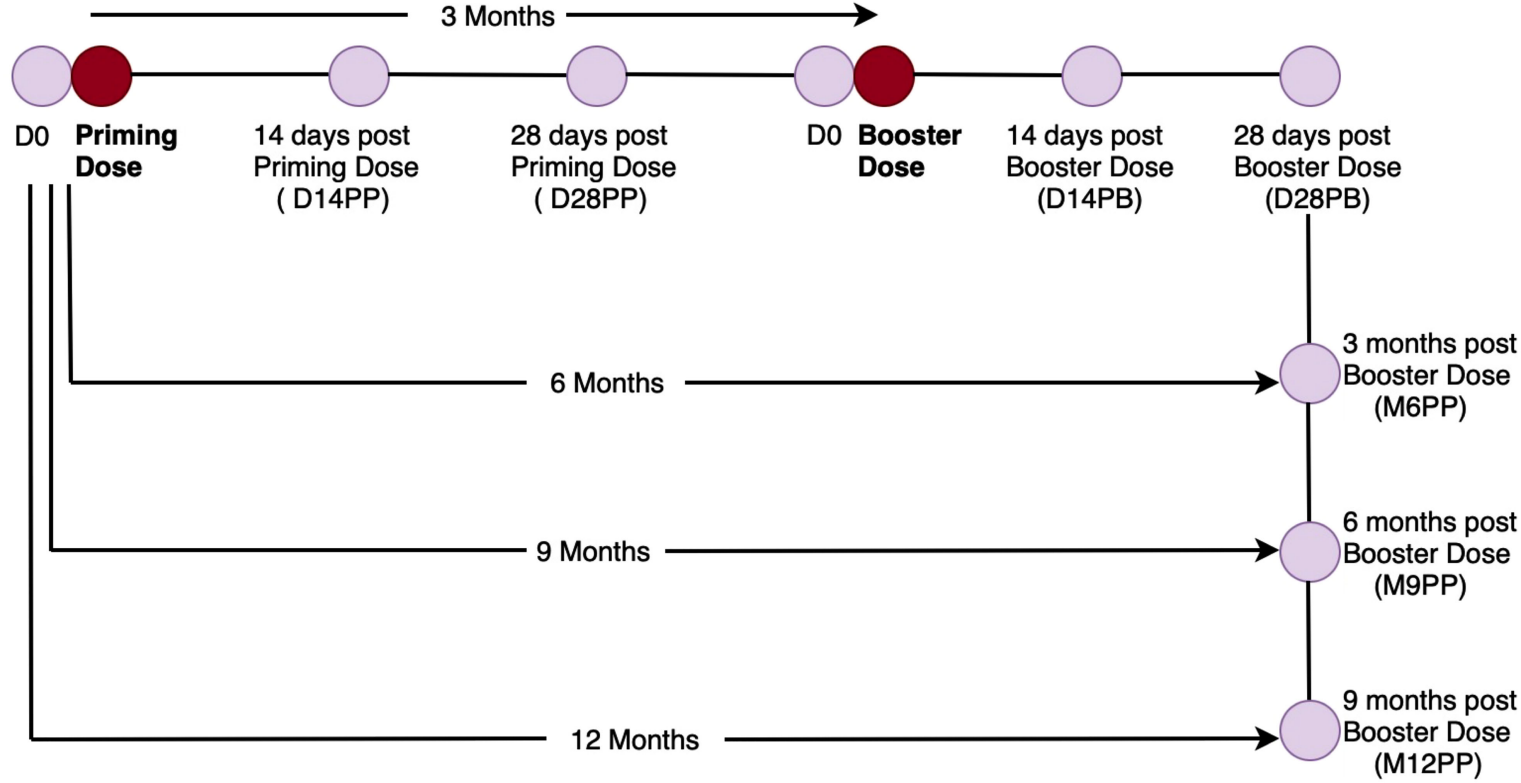
Graphical Illustration of the Cohort Time Points and Specimen Collection. **Figure 1** summarises the vaccination and specimen collection time points, beginning shortly before priming and continuing for up to one year after the initial vaccination.

Day 0 specimens (n= 64) were obtained before or immediately after (0–7 days) receiving the priming dose, while day 14 post-prime (D14PP) specimens (n= 71) were obtained 12–16 days after vaccination. Day 28 post-prime (D28PP) specimens (n= 84) were collected 28 days following the initial vaccination. The specimens for the booster dose (n= 50) were collected 90 days, or approximately three months, after the initial vaccination. The day-14 post-booster (D14PB) specimens (n= 64) were collected between 12 and 16 days after the booster dose. Post-booster day 28 (D28PB), specimens (n= 66) were collected 26 to 30 days after the second dose. Six months (168–192 days) after the priming dose, or three months after the second vaccination, specimens (n= 75) for the sixth month (M6PP) were collected. The month 9 samples (M9PP) were collected nine months after the first vaccination or six months after the second vaccination (n= 33). The month-12 samples (M12PP) were collected 12 months after the initial vaccination or nine months after the second injection (n= 12), as summarised in **Figure 1**. The days between the PCR or admission date and the vaccination date ranged from 52 to 557, with a median (IQR) of 286 (220–334) days. 75% of the subjects were vaccinated 334 days after infection (roughly a year after infection).

### Conventional in-house ELISA for detection of anti-SARS-CoV-2 IgG, IgM, and IgA binding antibodies

Spike- and nucleoprotein-directed IgG, IgM, and IgA levels were detected using a locally validated in-house ELISA, as previously described (23). Seropositivity cut-off OD values for IgG, IgM, and IgA for the spike protein were 0.432, 0.459, and 0.226, while the corresponding values for the nucleoprotein were 0.454, 0.229, and 0.225. Controls included predetermined negative and positive plasmas, the monoclonal antibodies CR3009 (2 mg/ml) for N or CR3022 (0.1 mg/ml) for S, and duplicate sets of blank wells. The plates were read at 450 nm using BioTek GEN5 software and a BioTek ELx808 microplate reader. The net OD response was derived by subtracting the blank wells’ OD values from the samples’ OD values.

### Estimating the levels of binding IgG, IgM, and IgA antibody concentrations

Antibody concentrations were estimated using the validated indirect ELISA by binding purified human IgG (Sigma, #12511), IgM (Sigma, # 18260), or IgA (Sigma, #12636) commercial antibody standards to anti-human kappa and lambda capture antibodies (Southern Biotech, #2060-01, #2070-0), as previously described (23). Standards were serially diluted and incubated in wells pre-coated with 50 μl of anti-human kappa and lambda at pre-specified dilutions before being subjected to the ELISA procedure described above. Using the BioTek GEN5 software, the OD450 values of the standards were used to generate non-linear, 4-parameter logistic (4-PL) standard curves. Antibody concentrations were extrapolated using the best linear range fit of the standard curves and then corrected for the associated dilution factor. Concentrations below the detection limit were assigned a 0 ng/ml value.

### Statistical analysis

Descriptive analysis was used to generate summary statistics for continuous variables and frequencies for categorical variables. Individual profile plots were generated to investigate the evolution of SARS-CoV-2 antibody OD values (nm) and concentrations (ng/ml) per subject. The optical densities (OD) and concentrations of the antibodies were compared using boxplots, and differences in OD values and concentrations between adjacent time points were determined using the Wilcoxon rank sum test. Line graphs of median OD values and concentrations were plotted to examine the overall evolution of antibodies. In addition, fold changes were calculated to determine the extent of the difference between two time points.

## Results

### Within two weeks of priming, vaccination substantially increased the prevalence of spike-directed antibodies

**Figure 2:**
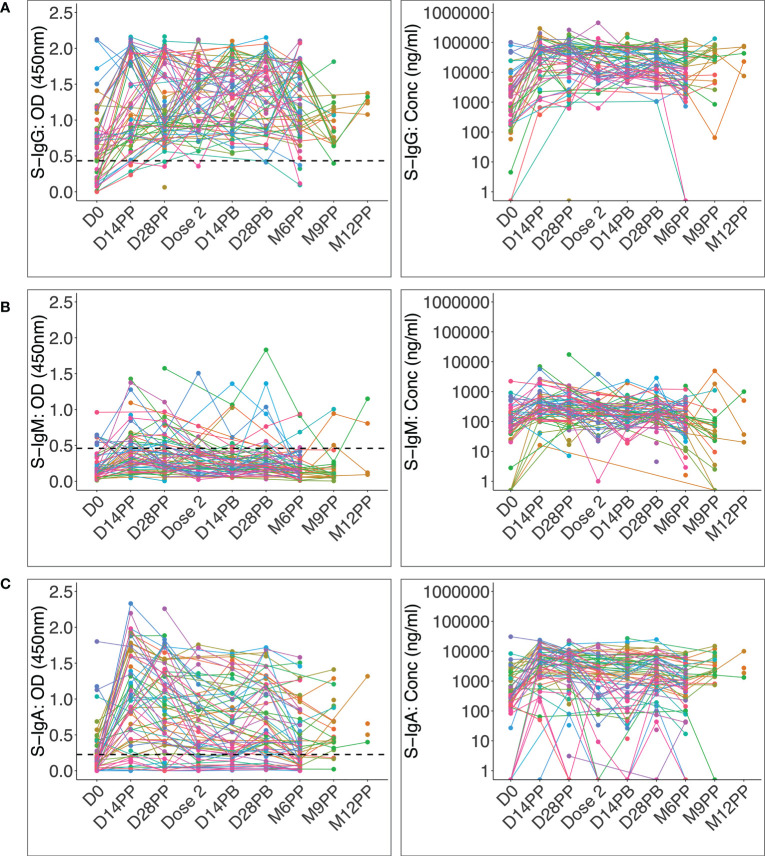
Individual profile plots of spike-directed IgG, IgM and IgA antibody optical densities and concentrations after vaccination. **Figure 2** shows the individual profile plots of spike-directed IgG **(A)**, IgM **(B)**, and IgA **(C)** antibody OD values (nm) and concentrations (ng/ml) over 12 months since the initial vaccination of individuals previously infected with mild or asymptomatic COVID-19. The dashed horizontal lines represent the cut-off points for antibody OD value seropositivity. The cut-offs for spike-directed IgG, IgM, and IgA seropositivity were 0.432, 0.459 and 0.226, respectively.

**Figure 3:**
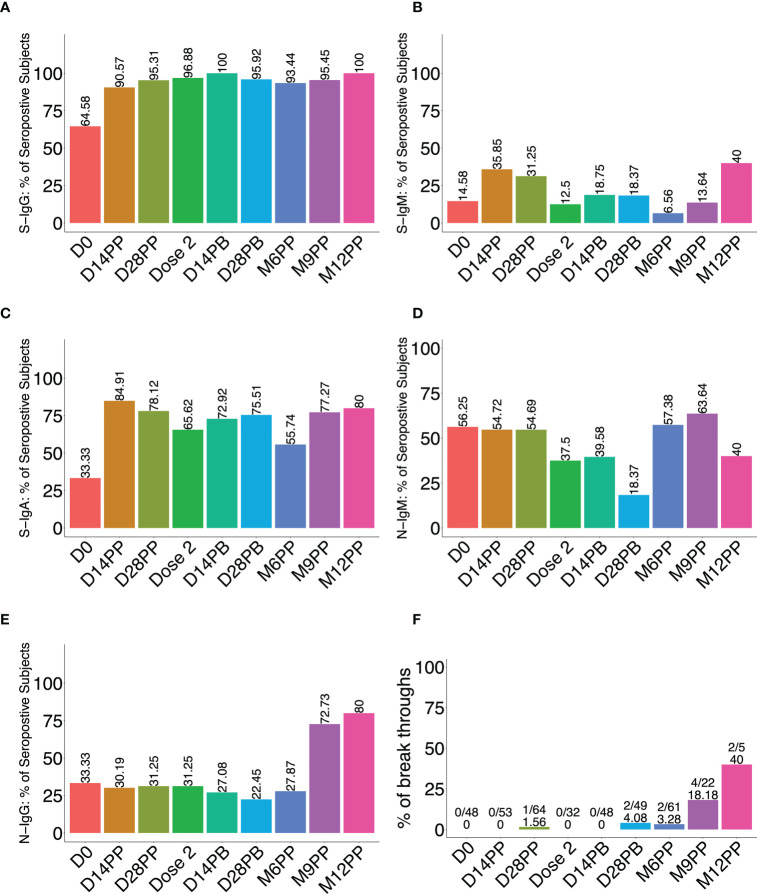
Percentage of seropositive participants following vaccination. **Figure 3** illustrates the proportion (%) of participants with spike-directed IgG **(A)**, IgM **(B)**, IgA **(C)**, and anti-nucleoprotein IgM **(D)** ad IgG **(E)** antibodies at various follow-up time points after vaccination (x-axis). **(F)** is a bar graph depicting the proportion of breakthrough subjects, defined as those who experienced an 11-fold increase in the concentration of nucleoprotein-directed IgG antibodies at each time point.

Administration of the AstraZeneca vaccine to convalescent Ugandans previously infected with rt-PCR-confirmed mild or asymptomatic COVID-19 led to a significant increase in the proportion of participants with spike-directed antibodies within two weeks of the first vaccine dose. This was especially notable for S-IgG and S-IgA, which remained elevated throughout the 12-month study period (**Figure 2**). At administration of the priming dose, 64.6% of participants possessed spike-directed IgG antibodies, indicating a substantial pre-existing robust virus-specific anti-spike antibodies at baseline, strengthened by vaccination. After the priming dose, the seroprevalence rose to 90.6% and 95.3% at 14 and 28 post-prime and then 96.9% before administering the booster dose. 100% of vaccinated individuals developed S-IgG antibodies two weeks after boosting (**Figure 3A**). One of the significant findings of previous studies with mRNA vaccines was that a longer duration between the time of initial infection and priming-dose vaccination significantly correlated with a more robust antibody response (24, 25). We did not find any correlation between duration between initial infection and AstraZeneca vaccination, and the magnitude of the peak IgG antibody response.

S-IgM seroprevalence changed marginally after the initial vaccination and barely after the booster (**Figure 3B**), rising from 14.6% at the start of the study to 35.9% two weeks after priming, 31.3% after 28 days, and 12.5% at the time of the booster dose. After boosting, the S-IgM antibody prevalence increased marginally to 18.8%, where it remained from 14 to 28 days. Boosting had a lower effect on S-IgM antibody production than priming. The modest change in IgM levels following vaccination of this previously infected cohort is not surprising. This is likely because the immune system is already primed to respond to the vaccine, and the memory B cells can quickly switch to producing IgG upon re-exposure to the vaccine (26, 27). Therefore, a booster vaccination would be expected to stimulate the production of IgG antibodies rather than IgM, resulting in a modest change or no change in IgM levels. The initial change in S-IgA prevalence was quite pronounced, increasing from 33.3% to 84.9% within two weeks of priming. After the boost, the seropositivity rate rose from 65.6% to 72.9% within two weeks; by the 28th day, it had reached 75.5% (**Figure 3C**). These data show that vaccination of COVID-19 convalescent Ugandans with the AstraZeneca vaccine induced an immune response characterised by a significant increase in the prevalence of spike-directed antibodies, highlighting the value of vaccination as an effective method for inducing immunity in individuals who have previously recovered from the disease.

### Six months after the initial vaccination, a rise in nucleoprotein seroprevalence, indicative of breakthroughs, emerged

Breakthrough subjects were defined as those who became re-infected at least three months after receiving both vaccine doses. Re-infection was deduced from the fold change in N-IgG concentration (ng/ml) between two consecutive time points and was previously defined for this population as a fold change of 11 or greater in N-IgG concentration (6). A subject with a fold change of 11 or higher was regarded as a breakthrough subject. Individual anti-nucleoprotein antibody profile plots showed some level of baseline N-IgG and N-IgM seropositivity (**Supplementary Figure 1**) at a prevalence of 33.3% and 56.3%, respectively. The prevalence of nucleoprotein antibody seropositivity was predominantly low but showed an increase of N-IgM to 57.4% by six months then to 63.6% and 72.7% by the ninth month, IgM and IgG respectively, indicating the possibility of reinfection (**Figures 3D, E**). Eight subjects were presumed breakthroughs (**Figure 3F**). The findings underscore the importance of administering two doses to maintain the desired level of protective immunity.

### Vaccination generated a superior spike-directed antibody response with differentially evolving IgG, IgM, and IgA levels

**Figure 4:**
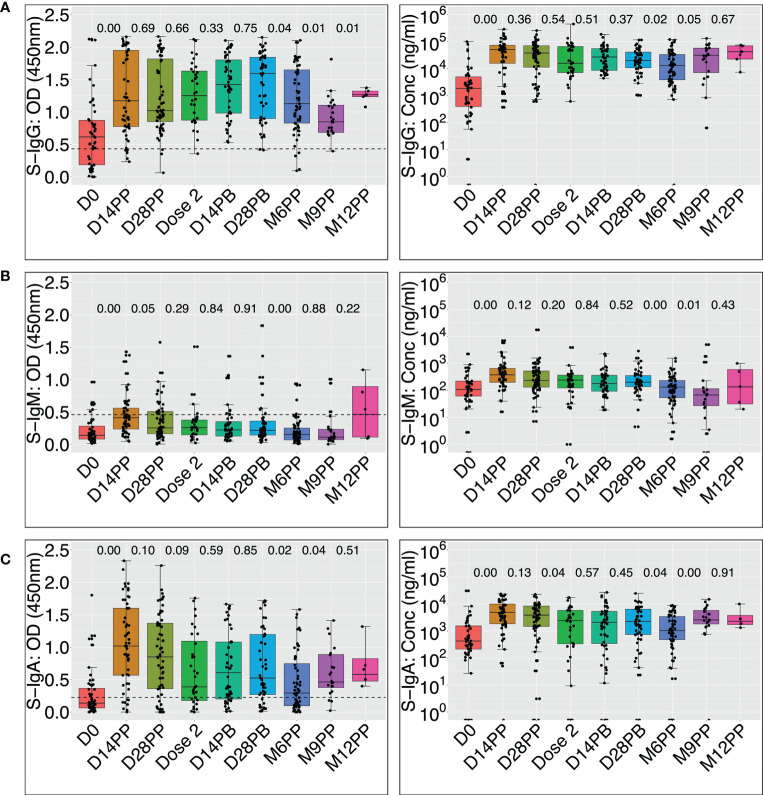
Box plots comparing vaccine-induced median spike-directed IgG, IgM and IgA antibody levels over time **Figure 4** uses the Wilcoxon signed rank test to compare median OD values at 450 nm and concentrations (ng/ml) of the spike-directed IgG, IgM, and IgA antibody levels across time points. OD at 450nm and concentrations are shown for IgG **(A)**, IgM **(B)**, and IgA **(C)**. P-values for differences between OD values or concentrations across adjacent time points are indicated. Horizontal dashed lines indicate cut-offs for the respective antibody isotypes. P values of 0.05 or less are considered statistically significant. The cut-offs for spike-directed IgG, IgM and IgA seropositivity were 0.432, 0.459 and 0.226, respectively.

The levels of spike-directed antibody concentrations significantly increased after AstraZeneca vaccination (**Figure 4** and **Table 1**), with IgG rising from a baseline median (IQR) of 35.6 (7.7 - 95.6) BAU/mL to a peak of 966.1 (305.9 - 1394.5) BAU/mL within two weeks of priming, Wilcoxon signed rank test (p < 0.0001). Afterwards, these levels progressively decayed to 299.3 (135.5 - 1252.2) BAU/mL by the time of boosting. Two weeks after boosting, the concentration of S-IgG rose to 510.9 (162.6 - 1043.6) BAU/mL before declining markedly to 245.1 (72.9 - 637.1) BAU/mL at six months since the initial dose. Taken together, these data show that vaccination elicited a robust S-IgG-directed humoral response that lasted at least six months post-priming before it started declining.

**Table 1:** Shows median antibody levels at different time points.

Time Point	Antibody	Median OD (IQR)	Median Conc (IQR) ng/mL	Median Conc (IQR) BAU/mL
D0	S-IgG	0.6153 (0.1855, 0.8718)	1896.15 (407.050, 5098.975)	35.5934 (7.7041, 95.5791)
	S-IgM	0.1410 (0.0818, 0.2823)	108.70 (61.55, 220.20)	4.47582 (2.7361, 8.5898)
	S-IgA	0.1375 (0.0625, 0.3653)	425.70 (213.4, 1544.3)	81.2093 (40.6916, 294.6952)
	N-IgG	0.2753 (0.1961, 0.5290)	1541.65 (313.95, 4992.23)	18.2142 (3.9103, 58.4169)
	N-IgM	0.3618 (0.1513, 0.6723)	388.35 (150.88, 806.90)	237.133 (91.0206, 494.6550)
D14PP	S-IgG	1.1730 (0.7760, 1.9515)	51578.75 (16330.45, 74450.35)	966.099 (305.9334, 1394.4610)
	S-IgM	0.4125 (0.2370, 0.5600)	377.00 (197.30, 652.20)	14.3753 (7.7449, 24.5293)
	S-IgA	1.0180 (0.5680, 1.6005)	4872.25 (1847.83, 9979.88)	929.838 (352.6233, 1904.6330)
	N-IgG	0.2075 (0.1055, 0.5115)	1212.50 (448.20, 3610.30)	14.3793 (5.4744, 42.3161)
	N-IgM	0.2490 (0.1410, 0.4285)	277.80 (178.50, 538.90)	169.114 (108.0175, 329.7619)
D28PP	S-IgG	1.0205 (0.8518, 1.8179)	38275.0 (11389.15, 71291.60)	716.933 (213.3878, 1335.3010)
	S-IgM	0.2550 (0.1650, 0.5065)	232.50 (132.25, 523.25)	9.04367 (5.3447, 19.7715)
	S-IgA	0.8500 (0.3585, 1.3698)	3856.10 (1470.28, 8435.30)	735.905 (280.5675, 1609.849)
	N-IgG	0.3368 (0.1543, 0.5428)	1881.15 (830.80, 3633.85)	22.1697 (9.9321, 42.5904)
	N-IgM	0.2478 (0.1296, 0.3970)	337.10 (144.30, 588.55)	205.600 (86.9751, 360.3102)
Dose 2	S-IgG	1.2510 (0.8735, 1.6308)	15972.35 (7228.85, 66855.00)	299.227 (135.4696, 1252.2080)
	S-IgM	0.2595 (0.1475, 0.3769)	240.50 (124.60, 365.63)	9.33885 (5.0625, 13.9556)
	S-IgA	0.3880 (0.1768, 1.0930)	2432.25 (333.675, 5774.212)	464.161 (63.6463, 1101.978)
	N-IgG	0.2240 (0.1330, 0.4627)	839.00 (353.30, 2181.80)	10.0276 (4.3687, 25.6726)
	N-IgM	0.2070 (0.1454, 0.3270)	351.15 (170.425, 462.325)	214.244 (103.0492, 282.6473)
D14PB	S-IgG	1.4248 (0.9823, 1.8030)	27275.1 (8678.6, 55718.5)	510.916 (162.6219, 1043.632)
	S-IgM	0.2238 (0.1280, 0.3521)	180.250 (93.0, 362.4)	7.11580 (3.89654, 13.83659)
	S-IgA	0.6068 (0.2000, 1.0806)	2091.70 (346.95, 5315.90)	399.167 (66.1798, 1014.509)
	N-IgG	0.2230 (0.1158, 0.4698)	606.50 (250.6, 2265.7)	7.31878 (3.172191, 26.65012)
	N-IgM	0.1843 (0.1205, 0.3173)	323.65 (129.125, 630.050)	197.324 (77.63838, 385.844)
D28PB	S-IgG	1.5970 (0.8980, 1.8440)	20259.0 (10813.97, 41475.75)	379.511 (202.6153, 776.8798)
	S-IgM	0.2170 (0.1440, 0.3610)	200.700 (137.875, 355.550)	7.87035 (5.552293, 13.58385)
	S-IgA	0.5250 (0.2690, 1.1965)	2262.90 (735.000, 6401.125)	431.841 (140.2395, 1221.625)
	N-IgG	0.1890 (0.0865, 0.3625)	388.700 (124.25, 1809.50)	4.78119 (1.700087, 21.33493)
	N-IgM	0.1230 (0.0730, 0.2180)	151.500 (95.60, 244.70)	91.4051 (57.01135, 148.7486)
M6PP	S-IgG	1.1280 (0.8280, 1.6490)	13081.1 (3886.00, 34012.95)	245.076 (72.86132, 637.109)
	S-IgM	0.1530 (0.0690, 0.2535)	131.900 (53.83333, 232.000)	5.33183 (2.451407, 9.025223)
	S-IgA	0.2928 (0.0970, 0.7460)	1038.70 (479.25, 3428.55)	198.201 (91.42938, 654.3062)
	N-IgG	0.1710 (0.0860, 0.4835)	959.750 (421.575, 3222.175)	11.4345 (5.16422, 37.79401)
	N-IgM	0.2470 (0.1465, 0.3895)	406.800 (241.150, 1267.867)	248.484 (146.5643, 778.2756)
M9PP	S-IgG	0.8480 (0.6833, 1.1049)	31736.2 (7359.00, 58151.98)	594.468 (137.9071, 1089.209)
	S-IgM	0.1120 (0.0728, 0.2340)	67.7000 (27.400, 115.900)	2.96305 (1.476097, 4.741481)
	S-IgA	0.4635 (0.3765, 0.8864)	2577.60 (1835.50, 5784.85)	491.902 (350.2711, 1104.008)
	N-IgG	0.5970 (0.3958, 1.1026)	2111.00 (790.85, 8392.90)	24.8477 (9.466643, 98.03813)
	N-IgM	0.2790 (0.2073, 0.3713)	303.650 (220.325, 409.375)	185.019 (133.7513, 250.0686)
M12PP	S-IgG	1.2660 (1.2350, 1.3230)	42862.9 (22729.5, 70612.6)	802.8597 (425.7812, 1322.584)
	S-IgM	0.4630 (0.1128, 0.8925)	268.750 (32.425, 625.725)	10.38119 (1.661504, 23.55249)
	S-IgA	0.5800 (0.4768, 0.8225)	2310.00 (1733.40, 4558.975)	440.8299 (330.7852, 870.049)
	N-IgG	0.8085 (0.6210, 0.9980)	19647.85 (5929.35, 37718.30)	229.1696 (69.33531, 439.7084)
	N-IgM	0.2330 (0.1520, 0.6150)	164.1500 (102.9, 651.6)	99.18831 (61.50284, 399.1031)

While most participants’ S-IgM remained below the cut-off, the median OD values significantly rose from a modest baseline of 0.14 (0.08 - 0.28) nm to 0.41 (0.24 - 0.56) nm 14 days later (**Figure 3B**); Wilcoxon signed rank sum test (p < 0.0001). Corresponding concentrations also changed from 4.5 (IQR: 2.7 - 8.6) BAU/mL to 14.4 (IQR: 7.7 - 24.5) BAU/mL on day 14 (p<0.0001) before dropping to 9.0 (IQR: 5.3 - 19.8) BAU/mL by day 28 after priming (**Figure 3B**), indicating a rise in S-IgM humoral immunity to SARS-CoV-2. These findings support using vaccination as a SARS-CoV-2 prevention measure, even in people with a mild or asymptomatic infection history. The data suggest that using IgM as a proxy for early antibody responses to vaccination of previously infected individuals may underestimate the levels of antibody response mediated by the vaccine. The data show that vaccination can enhance the humoral immune response of previously infected individuals and that the antibody response mediated by the vaccine may exceed what can be measured by IgM.

The baseline S-IgA concentration was modest at 81.2 (40.7 - 294.7) BAU/mL in 31% of the participants; this increased significantly to 929.8 (352.6 - 1904.6) BAU/mL in 85.2% of the participants (p < 0.0001) two weeks after priming. Corresponding OD values increased from a baseline of 0.14 (IQR: 0.06 - 0.37) nm to a maximum of 1.02 (0.57–1.60) nm 14 days after the initial vaccination (p < 0.0001, **Figure 3C**). This robust vaccine-induced S-IgA in a population previously demonstrated to lack an IgA response to natural infection (6) suggests a qualitatively superior memory response to the vaccine compared to the response induced by the primary natural infection. Although the vaccine elicited a robust S-IgA response, it was not sustained and had decreased significantly to near the threshold by the time of boosting.

Regarding nucleoproteins, these subjects’ N-IgG and N-IgM antibody levels were low, decreased over time, and stayed below the cut-off in most subjects until nine months, when they began to rise, suggesting the possibility of re-infection (**Supplementary Figure 2**). The findings imply that re-infection may be unlikely after the initial infection if antibodies are present but will likely occur when they significantly drop. Individuals who have recovered from an initial infection may still be vulnerable to subsequent infections if their antibody concentrations drop below a certain threshold. These findings demonstrate that the AstraZeneca vaccine successfully induced a humoral immune response in individuals with previous mild or asymptomatic COVID-19 infections. The increase in anti-spike antibodies in all subjects after two vaccine doses shows a successful immune response; however, waning immunity six months after the priming dose emphasises the need for boosting six months after the priming dose. In addition, the results demonstrate that vaccination can produce a more robust and qualitatively superior immune response than natural infection. After priming, the dramatic increase in S-IgG and -IgA levels suggests that people with mild or asymptomatic COVID-19 infections can benefit from vaccination. These findings support the use of COVID-19 vaccines to protect against subsequent infections in previously infected individuals, even if they did not exhibit symptoms during primary infection.

### Vaccination resulted in an early and rapid rise in S-IgG and S-IgA levels and a longer S-IgG antibody durability

**Figure 5:**
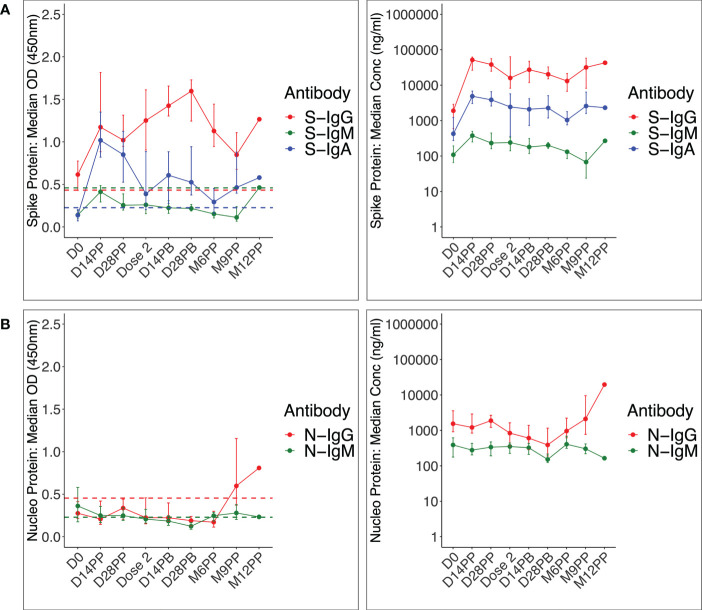
shows the median trajectories of IgG, IgM, and IgA at after vaccination (**Figure 5**) shows the median spike - **(A)** and nucleoprotein-directed **(B)** IgG, IgM, and IgA antibody OD values (m) and concentrations (ng/ml) over time, medians at a specific time point are plotted. The cut-offs for spike-directed IgG, IgM and IgA seropositivity were 0.432, 0.459 and 0.226, respectively. The cut-offs for nucleoprotein-directed IgG, IgM and IgA seropositivity were 0.454, 0.229 and 0.225, respectively.

Using median anti-spike antibody trajectories, IgG was the most induced antibody, followed by IgA, while IgM was barely detectable. Initial IgG levels were higher than the threshold and continued to rise until day 28 after the boost (approximately four months after the priming dose), when they started declining. The IgM antibody OD values were consistently lower than the cut-off level, whereas the S-IgA response rose quickly, peaked 14 days after priming, and remained above the cut-off level before declining over time (**Figure 5A**). Spike-directed IgG and IgA were the most prevalent Immunoglobulins induced by the vaccine, rapidly peaking fourteen days after the priming dose. However, neither N-IgG nor N-IgM was detectable before six months (**Figure 5B**), indicating no re-infection.

Vaccine-induced antibodies that were of significantly higher magnitude than in prior natural immunity (p > 0.05; **Supplementary Figures 3A, 3B**). These data suggest the importance of S-IgA and S-IgG as essential components of the humoral immunity against SARS-CoV-2 induced by vaccination in this population. The early and rapid increase in S-IgA antibodies in response to vaccination and the longer durability of S-IgG antibodies demonstrate the importance of both short-term and long-term humoral immunity induced by vaccination. S-IgA was essential in developing early humoral immunity against the SARS-CoV-2 virus, acting as a second line of defence alongside S-IgG antibodies. The results demonstrate the importance of S-IgA as a reliable predictor of the humoral immune response to the COVID-19 vaccination, particularly when measured in conjunction with S-IgG. The pattern of S-IgG and S-IgA levels observed in this study suggests that vaccinations of previously infected individuals stimulated an effective immune response. In addition, the results demonstrate that S-IgG and S-IgA antibodies are essential for detecting vaccination exposure and estimating the associated immunity.

### The fold change in vaccine-elicited spike antibodies was most pronounced two weeks after priming

**Figure 6:**
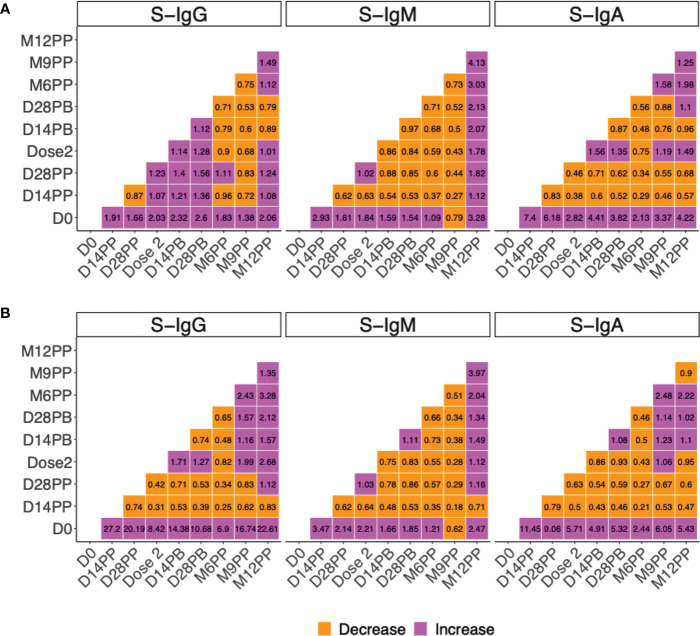
Median fold change in in spike-directed antibody levels over time. **Figure 6** summarises the fold change in spike-directed IgG, IgM and IgA antibody OD values **(A)** and concentrations **(B)** across the different specimen collection time points. The heat map shows the extent of change (increase or decrease) in vaccine-induced antibody response over time. The change between time points is shown with each cell indicating an increase or decrease between any two timepoints at the x and y axis. Fold change between 0 to 1 indicate a decrease (orange), Fold change of 1 indicates no change while fold change greater than 1 indicate an increase (purple).

The median change in magnitudes of vaccine-induced antibodies was established over time and summarised on heat maps (**Figure 6**). Levels of anti-spike IgA and IgG antibodies differed significantly before and after vaccination, indicating a substantial immune response due to vaccination. Even though the 1.9- and 1.7-fold increases in S-IgG OD values from baseline to 14 and 28 days were encouraging, the 7.4- and 6.2-fold increases in S-IgA were notable (**Figure 6A**). These findings demonstrated the vaccine’s effectiveness in priming S-IgA responses, which are critical in protecting against respiratory tract infections. Correspondingly, concentrations of S-IgA and S-IgG rose by 11.5- and 27.2-fold on day 14 and 9.1 and 20.2 times higher 28 days after priming, respectively (**Figure 6B**). At all subsequent visits, median S-IgG and S-IgA concentrations remained at least three-fold higher than baseline. The higher concentrations of S-IgG overall imply that S-IgG concentrations may be more effective at inducing long-term protection against infection. These findings suggest that S-IgG concentrations should be tracked over time to assess vaccine effectiveness and provide valuable insight into the success of vaccination campaigns. There was minimal change in spike-directed antibody concentrations after the boosting, with only a 1.71-, 0.75-, and 0.86-fold change in IgG, IgM, and IgA antibody concentrations 14 days after the boost and a 1.27-, 0.83-, and 0.93-fold change 28 days after boosting, respectively. Anti-nucleoprotein antibody concentrations changed very little over time (**Supplementary Figure 4**). However, a significant increase in N-IgG from month 9 indicated the possibility of re-infection. This finding is significant because it suggests that, although the initial infection may result in long-term immunity against re-infection, individuals must maintain adequate antibody levels to maintain this protection. By this, S-IgA and S-IgG antibodies were significantly elevated at month 12, indicating a heightened immune response in the presence of potentially infectious antigens. These findings demonstrate that re-infection after vaccination is possible and emphasise the significance of maintaining a robust immune memory.

## Discussion

The timing of booster vaccinations and the chronology and duration of immunity from SARS-CoV-2 infection and vaccination are essential factors that shape pandemic policy interventions. Two years after the initial shipment of COVID-19 vaccines to Africa via the COVAX facility on February 24, 2021, there is still a significant lack of systematic data regarding the evolution of the sub-Saharan African population’s immunity to the vaccines, despite the availability of extensive data from other regions. This knowledge is critical for informing evidence-based policies and practices that will effectively address the pandemic in this region. This study investigated the development, specificity, and durability of SARS-CoV-2-specific IgG, IgM, and IgA antibody responses to AstraZeneca vaccination in a Ugandan population with rt-PCR-confirmed prior infection. We aimed to examine how the immune responses of 86 individuals with prior COVID-19 infection evolved following two vaccination doses. The study’s significance lies in providing the first essential data on the timeline of vaccine-elicited antibody kinetics in a Sub-Saharan African setting in a population with rt-PCR-confirmed prior mild or asymptomatic infection. Anti-spike antibody seropositivity was 65% for IgG and 33% for IgA at baseline. Within two weeks of receiving the priming dose, the seroprevalence increased to 91% and 85%, respectively. Whereas boosting had a significantly smaller effect on the spike antibody levels, it increased the overall spike-directed seropositivity, achieving a 100% success rate within two weeks of the boost. Correspondingly, within two weeks of vaccination, concentrations of S-IgA and S-IgG increased 11- and 27-fold, respectively. These increases persisted 28 days after priming, with S-IgA and S-IgG concentrations remaining nine- and 20-fold higher than baseline, respectively. Antibodies directed against nucleoproteins remained low for nine months before rising, indicating the possibility of reinfection. This study provides valuable data on the evolution and persistence of vaccine-induced antibodies in a sub-Saharan African population with prior COVID-19 infection. These findings can inform evidence-based policies and practices that will effectively address the pandemic in this region.

Infection with and vaccination against SARS-CoV-2 induces functional immune memories that enhance disease protection, a phenomenon known as “hybrid immunity” (28, 29). We showed that vaccination of individuals that recovered from mild or asymptomatic infection elicited rapid and robust antibody responses against the virus that peaked within two weeks of priming and were marked by a significant increase in seroprevalence and levels of spike-directed antibodies. This was especially true for S-IgG and S-IgA, which peaked at a geometric mean concentration (95% CI) of 518.71 (518.69–518.54) BAU/mL and remained elevated throughout the entire 12-month study period, demonstrating the vaccine’s effectiveness in priming a robust and long-lasting memory response. In contrast, after priming, peak levels of S-IgM antibodies were significantly lower at 19.46 (CI 19.43–19.49) BAU/mL, declined rapidly, and remained low for the more significant part of the 12-month study period. These data agree with the more extended durability of IgG and IgA relative to IgM antibodies (4), and the vaccine-induced expansion of S-IgG and IgA levels (5, 30) demonstrated by other vaccines. In contrast, the Oxford-AstraZeneca vaccine elicited a more rapid immune response in this cohort than in two African studies (29, 31), but agreed with an Israeli study where the peak occurred before three weeks (32).

The varying times for the peak of antibodies after priming may be attributable to the design of the studies. For instance, the two African studies only examined antibody levels one month after priming, missing a possible peak at two weeks. Using the same vaccine formulation, Thai researchers noted a dose-dependent antibody boost (33), contrasting starkly with our findings, which revealed the highest antibody responses to form after the priming dose and minor change after the booster dose. This disparity could be explained by factors that affect vaccine effectiveness, such as age, gender, pre-existing immunity, and unknown environmental factors (34, 35). These data highlight the importance of generating population-based data to guide local vaccination strategies. There is a need for research to further understand how different populations respond to vaccines, how potential confounders may alter vaccine efficacy, and how this can be used to improve and maximise vaccination strategies.

The anti-spike antibody levels in this study are comparable to the 588.0 U/mL (IQR, 250.0-2500.0 AU/mL) seen in previously infected and then vaccinated Indians who received the same vaccine (36), but they are significantly higher than the 112.3 BAU/mL (61.7-204.4) observed in a clinical trial for infection-naive subjects in South Africa 28 days after priming (37). Therefore, this study provides evidence that subjects with prior exposure to SARS-CoV-2 responded better to the vaccine and developed higher levels of anti-spike antibody concentrations than those without prior exposure. Those with pre-existing immunity to spike proteins may be more likely to respond favourably to vaccination. This is consistent with the knowledge that pre-existing immunity is more likely to contain memory B cells and plasma cells and is better equipped to rapidly respond when presented with a vaccine antigen than infection-naive B cells (38, 39). Hybrid immunity was found to be robust in this study, which is not surprising given that previous studies have reported similar findings with AstraZeneca (36, 40) and others showed prior infection to provide 80–95% protection against symptomatic COVID-19 reinfections for at least eight months (28, 41–43). These are consistent with our findings that prior infection provides 93% anti-spike seropositivity coinciding with absence of symptomatic COVID-19 reinfection for at least six months. The study’s results echo the outcomes of earlier studies (44, 45), which indicated that individuals who elicit hybrid immunity are highly likely to be protected from reinfection for up to eight months. Similar drops in immunity have been observed six months after the priming dose in Asia (46) other low- and middle-income countries (LMICs) with similar sociodemographic characteristics (47) and high-income countries (43). The fact that the decline in immunity occurs in such a wide variety of settings suggests that it is a phenomenon that is independent of geographical factors. As in other studies (48), a priming dose after infection improved the virus-specific immunity, highlighting the need for a suitable boosting strategy.

There were some limitations to this study. First, the study could only assess the presence of antibodies, not their function. As a result, the findings should be interpreted with caution as they offer limited insight into the potential impact of antibody-mediated protection. As the study lacked information on antibody functionality or its role in protection against disease, reinfection, or transmission of SARS-CoV-2 to other individuals, additional research is necessary to investigate these outcomes. It did, however, indicate that a substantial proportion of the population has developed detectable levels of antibodies and provides some insight into the possibility of protective immunity against SARS-CoV-2. In addition, this study could not determine whether antibodies were associated with a change in symptom severity or with protection against reinfection or transmission of SARS-CoV-2 to other individuals; thus, additional research is required to investigate these outcomes. In order to reliably inform policy, it is also necessary to assess the reinfection rates in the presence of circulating antibodies and identify risk factors for antibody loss.

Another limitation is keeping track of the virus’s rapidly evolving genome. While the predominant virus at the time of infections in this cohort was A23.1, SARS-CoV-2 has evolved, with the Omicron variant having far more mutations in the spike than previously reported variants and circulating the most widely. The rapid evolution of SARS-CoV-2, especially with the emergence of the Omicron variant, poses a significant challenge for monitoring the virus and tracking the induced immune responses. The high mutation rate of Omicron’s spike protein may impede the detectability of spike-binding antibodies, resulting in an underestimation of the actual level of population immunity. This challenge highlights the need to develop new serological assays capable of detecting antibodies to the Omicron variant. These assays could target multiple regions of the spike protein or concentrate on regions of the spike protein that are less likely to undergo significant mutations. Alternately, other measures of the immune response, such as T-cell responses, could be included in monitoring efforts to provide a more thorough understanding of the immune response to SARS-CoV-2 infection or vaccination. In addition, continued genomic surveillance is required to monitor the evolution of the virus and inform public health strategies. Continued studies are needed to assess the effectiveness of existing vaccines and therapeutics against the Omicron variant and to develop new interventions capable of effectively addressing the virus’s rapid evolution.

Although we were able to detect antibody responses in a substantial proportion of participants at the 9-month and 12-month post-primary vaccination (PP) time points, the lower number of specimens evaluated at these later time points limits the statistical power of our analyses and makes it more difficult to draw robust conclusions from these time points. Nevertheless, we included these time points in our study to shed light on the durability of antibody responses to the AstraZeneca vaccine in previously infected individuals over an extended period. These findings demonstrate the need for additional research to substantiate our findings and provide more definitive conclusions. Additionally, this study was conducted to fit into the real-world vaccination policy in the country, and it was impossible to intentionally add investigations of a single-dose cohort. Adding a third cohort receiving only one injection could have provided valuable information on the optimal vaccination strategy for individuals previously infected with SARS-CoV-2. Specifically, this cohort could have helped to determine whether a single dose of the vaccine is sufficient to confer adequate protection in individuals with prior infection, given that their immune systems have already been primed against the virus. Furthermore, this third cohort could have provided real-world data on the proportion of individuals who did not get fully vaccinated, given that as of March 25, 2023, only 67% of Ugandans have received both doses of the vaccine. This information is crucial for informing public health policies and vaccine distribution strategies in Uganda and beyond.

Lastly, the population under consideration was relatively young and predominately male, despite age- and gender-specific correlations. Although the data is exceptionally accurate for the African continent, where an estimated 70% of the population is under 30 years old, it should be interpreted with caution as there may be limitations to the generalizability of the findings to other age groups and females. Concerning is the fact that the study’s age range may not provide adequate information on the elderly, who are at the highest risk for severe COVID-19 infection. Future research should include a more diverse demographic population spanning various age groups and genders to increase the external validity of the findings. Despite these limitations, the results of this study indicate that all individuals with a prior SARS-CoV-2 infection will develop spike-directed antibodies after receiving two doses of the AstraZeneca vaccine. These findings support proponents of routine booster shots to maintain the protective antibody levels necessary for longer SARS-CoV-2 immunity (49, 50). The induced immunity was rapid, robust, sustained, and peaked within two weeks, demonstrating the effectiveness of booster shots for long-term immunity. The lack of anti-nucleoprotein antibodies for nine months suggests that vaccine-induced antibodies protected against reinfection during this period.

In conclusion, the study found that administration of the AstraZeneca vaccine increased the spike-directed IgG, IgM, and IgA antibodies, resulting in a rapid and robust immune response against the virus in most participants two weeks after the initial dose and all participants four weeks after the initial dose. This finding is significant because it suggests that even those with asymptomatic infection can benefit from the vaccine. This study provides policymakers with evidence-based insight into the timing and duration of antibody-mediated immunity conferred by COVID-19 vaccination in a prior mild or asymptomatic sub-Saharan setting. As we face the rapid emergence and evolution of new variants, we must demonstrate these antibodies’ capacity to neutralise emerging viruses. The low levels of induced S-IgM suggest that using it as a surrogate for monitoring vaccine-induced responses would significantly underestimate induced immunity. This suggests that even those with prior asymptomatic infections can benefit from vaccination. The two AstraZeneca vaccine doses effectively induced short-term and long-term immunity and protected against the virus for at least nine months. The absence of anti-nucleoprotein antibodies until nine months, indicating the absence of infection, is an encouraging indication of the vaccine’s efficacy and suggests that vaccine-induced antibodies protected against reinfection during this time. Reinfections in individuals with hybrid immunity imply a potential need for further boosting. Our findings suggest that vaccination can protect against reinfection during this time and provide policymakers with evidence-based insights into the timing and duration of vaccine-induced immunity in this population. However, additional research is needed to understand the chronology of immunity conferred by vaccination in the context of variations in SARS-CoV-2, populations, and age groups. Monitoring anti-spike IgG and IgA when assessing vaccine-induced antibody responses is suggested for this population.

## Data availability statement

The original contributions presented in the study are included in the article/**Supplementary Material**. Further inquiries can be directed to the corresponding author.

## Ethics statement

The studies involving human participants were reviewed and approved by the Research and Ethics Committee of the Uganda Virus Research Institute (GC/127/833) and the Uganda National Council for Science and Technology (HS637ES). The patients/participants provided their written informed consent to participate in this study.

## Author contributions

Conceptualization and methodology: JSer. Laboratory investigation: CB, SM, GKO, LK, IK, JSem, GO, PE, AN, BG, and The COVID-19 Immunoprofiling team. Data curation, software, and formal statistical analysis: VA and JSer. Writing- original draft; JSer, GKO and VA. Writing- reviewing and editing; PK and MM. Funding acquisition; MM and JSer. Project administration: JSer and BA. Supervision; JSer, PK. All authors read and approved the final manuscript.

## Group members of the COVID-19 immunoprofiling team

Christine Hermilia Akoli, Deus Mwesigwa, Ivan Ssali, Hellen Nantambi, Angela Namuyanja, Solomon Opio, Arthur Watelo Kalyebi, Joseph Ssebwana Katende.
