# Supplementary material for: Seroprevalence and durability of antibody responses to AstraZeneca vaccination in Ugandans with prior mild or asymptomatic COVID-19: implications for vaccine policy
Source: Front Immunol. 2023 May 2;14:1183983. doi: 10.3389/fimmu.2023.1183983 (PMC10187141; doi:10.3389/fimmu.2023.1183983)
Supplement: Supplementary file 1 [file DataSheet_1.pdf]

Supplementary Material

Supplementary Figures

Supplementary Figure 1: *Individual profile plots of nucleoprotein-directed IgG and IgM antibody optical densities and concentrations after vaccination*

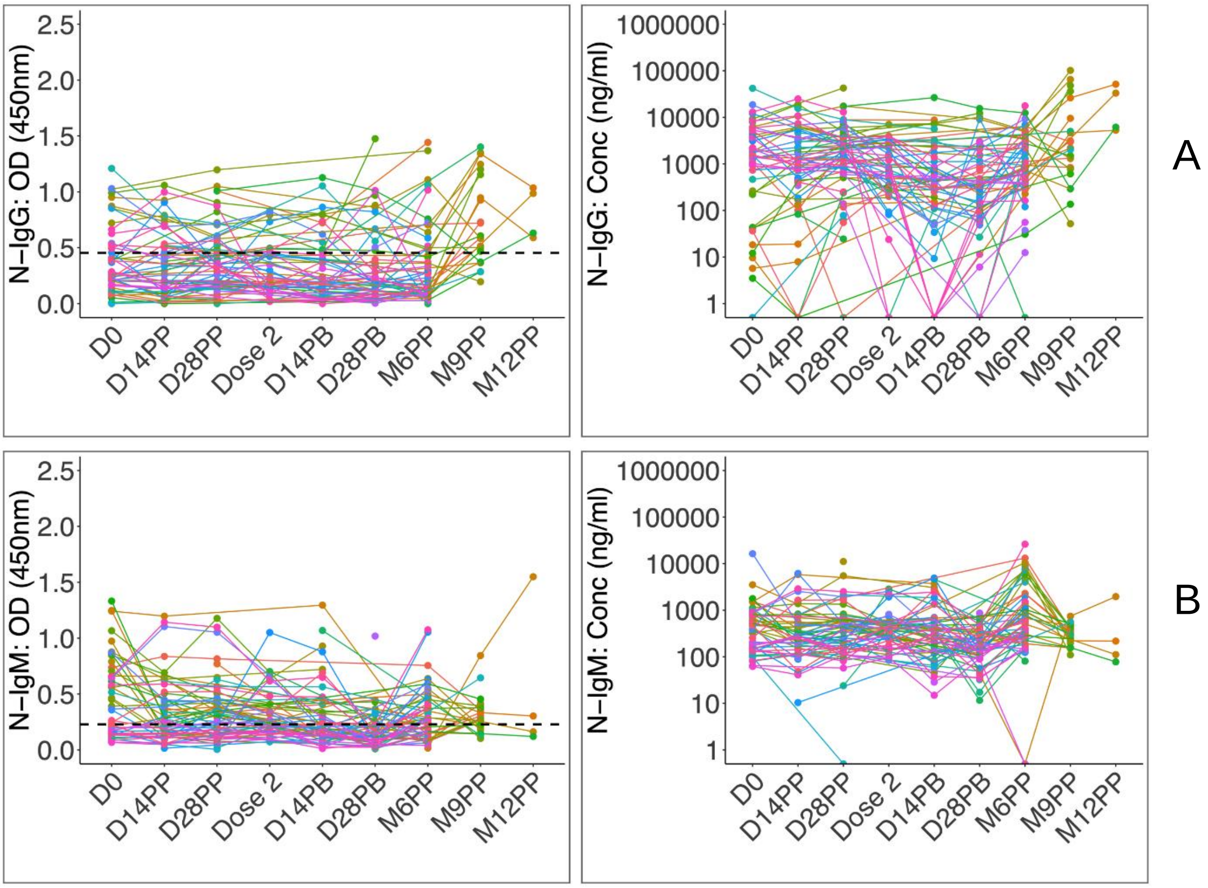

Supplementary Figure 1 *shows the individual profile plots of nucleoprotein-directed IgG and IgM antibody OD values (450nm) and concentrations (ng/ml) over 12 months since the initial vaccination of individuals that were previously infected with mild or asymptomatic COVID-19. The dashed lines represent the cut off points for antibody OD value seropositivity.*

Supplementary Figure 2*: Box plots comparing vaccine-induced nucleoprotein-directed IgG and IgM antibody levels over time.*

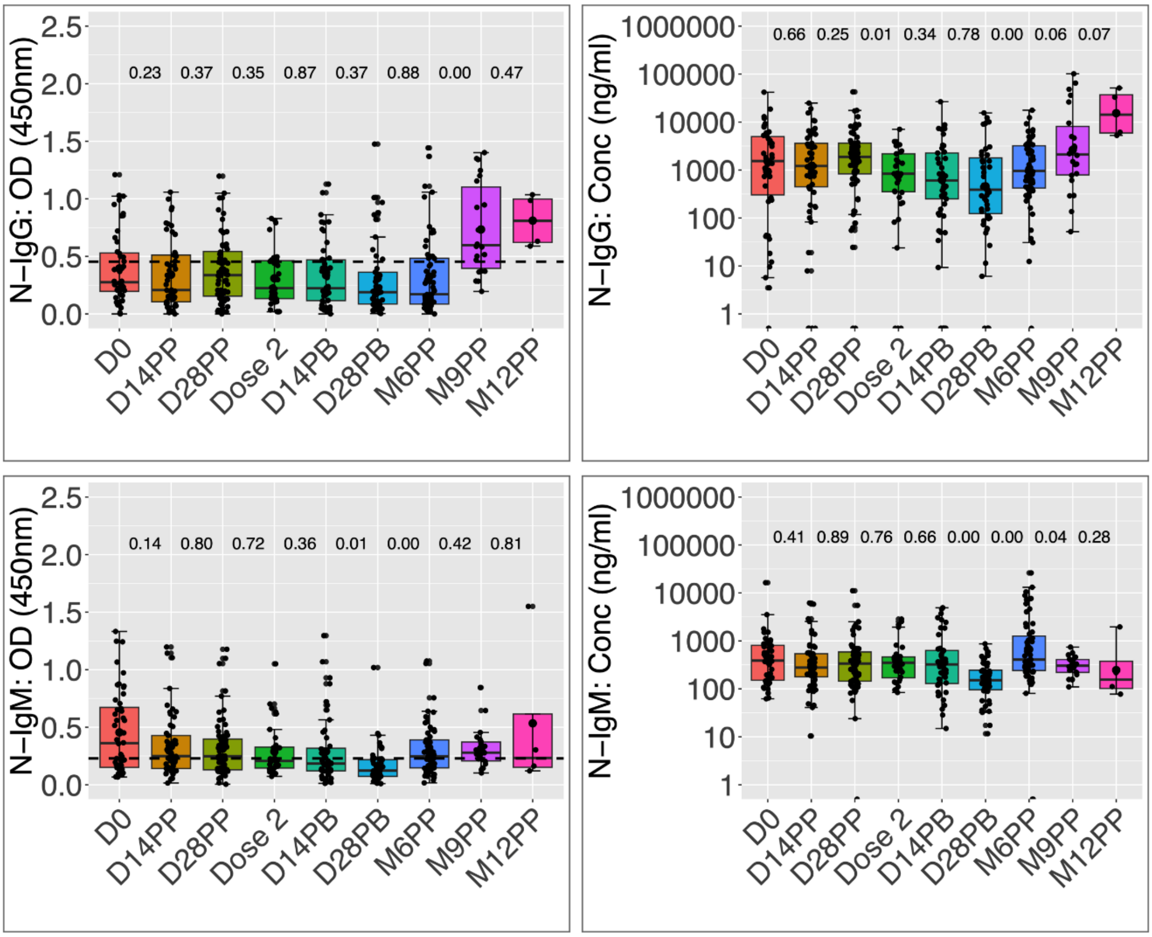

Supplementary Figure 2 *illustrates the use of the Wilcoxon signed rank test to compare the median OD levels and concentrations of nucleoprotein-directed IgG and IgM antibody levels over time, with the p-values for the difference between OD or concentration across adjacent time points indicated. Horizontal dashed lines denote the respective isotype cut-offs. The cut-offs for N-IgG and N-IgM are 0.454 and 0.229 respectively. P values of 0.05 or less are considered statistically significant.*

***Supplementary Figure 3** shows the pre- and post-vaccination IgG and IgM median trajectories over time*

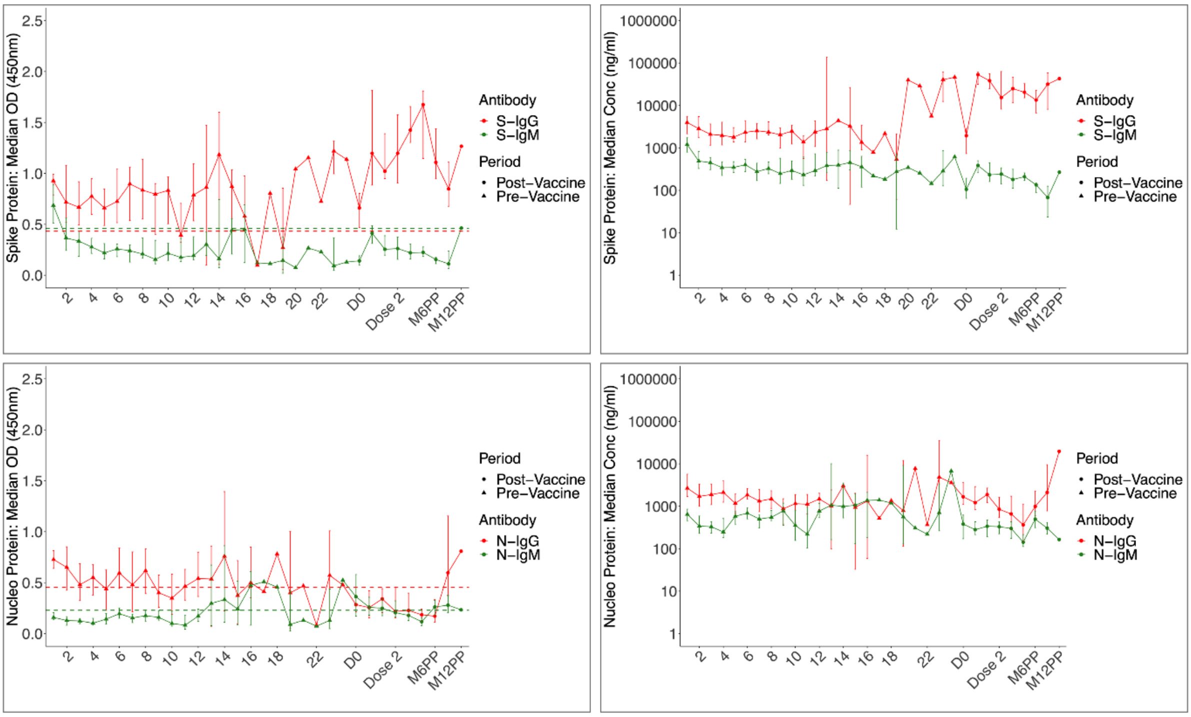

A

B

***Supplementary Figure 3** shows the median spike- (**A**) and nucleoprotein-directed (**B**) IgG and IgM antibody OD values (450nm) and concentrations (ng/ml) before and after vaccination; medians at specified time points are plotted.*

Supplementary Figure 4*: Median fold change in in nucleoprotein-directed antibody levels over time*

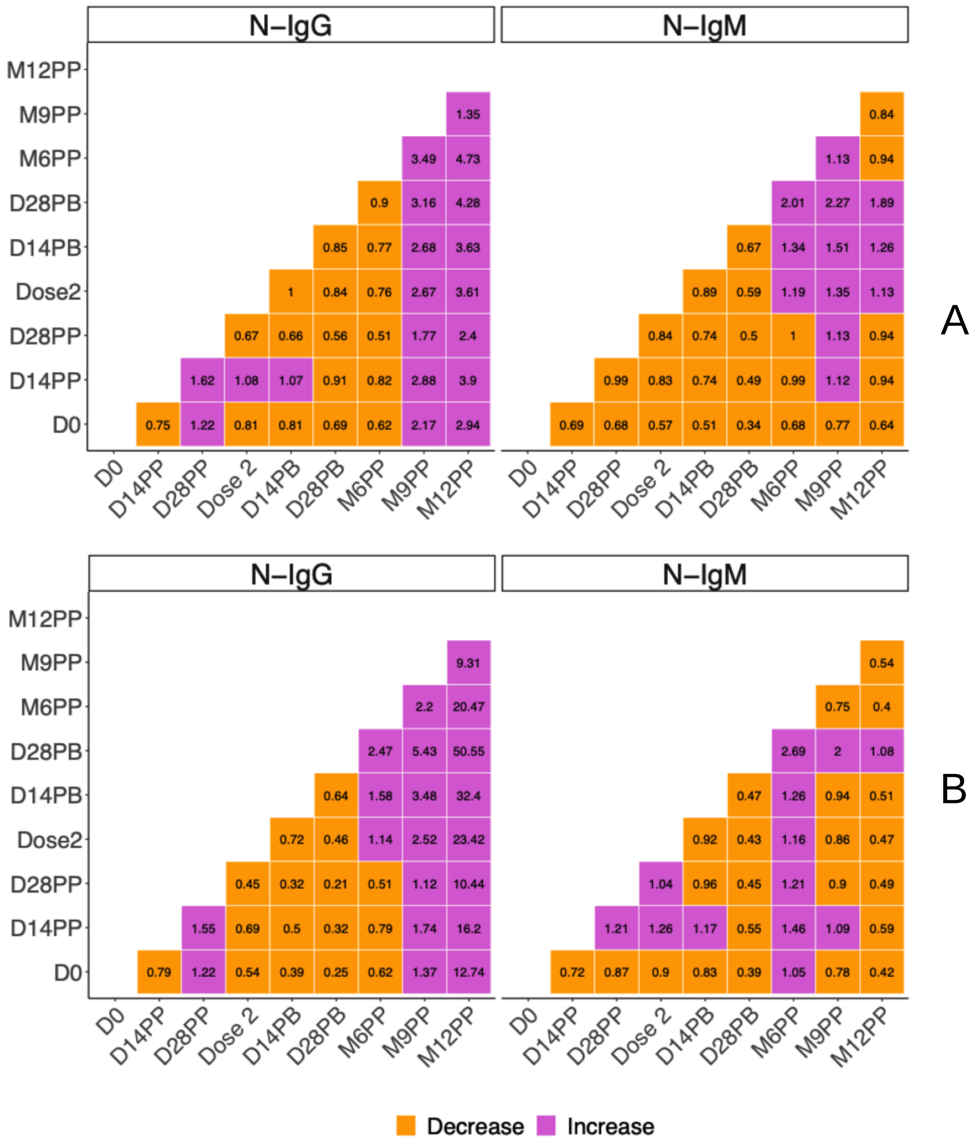

Supplementary Figure 4 *summarises the fold change in nucleoprotein-directed IgG and IgM antibody OD values (**A**) and concentrations (**B**) across the different specimen collection time points. The heat map shows the extent of change (increase or decrease) in vaccine-induced antibody response over time. The change between time points is shown with each cell indicating an increase or decrease between any two time-points at the x and y axis. Fold change between 0 to 1 indicate a decrease (orange), Fold change of 1 indicates no change while fold change greater than 1 indicate an increase (purple).*
